# Smoking cessation and vascular endothelial function

**DOI:** 10.1038/s41440-023-01455-z

**Published:** 2023-10-12

**Authors:** Yukihito Higashi

**Affiliations:** 1 Department of Regenerative Medicine, Research Institute for Radiation Biology and Medicine, Hiroshima University, Hiroshima, Japan; 2 Division of Regeneration and Medicine, Medical Center for Translational and Clinical Research, Hiroshima University Hospital, Hiroshima, Japan

**Keywords:** Smoking, Smoking cessation, Endothelial function, Oxidative stress, Rho-associated kinase

## Abstract

Smoking is associated with vascular endothelial dysfunction. It is thought that smoking impairs vascular endothelial function through a decrease in nitric oxide bioavailability induced by activation of oxidative stress and inflammation. Endothelial dysfunction can be improved or augmented by appropriate interventions including pharmacotherapy, administration of supplements and lifestyle modifications. Although there have not been many studies, the effects of smoking cessation on endothelial function have been shown. In those studies, it was shown that smoking cessation does not always have a positive effect on vascular endothelial function. In this review, I will focus on the role of smoking in endothelial function and the effects of smoking cessation on endothelial function.

**Figure:**
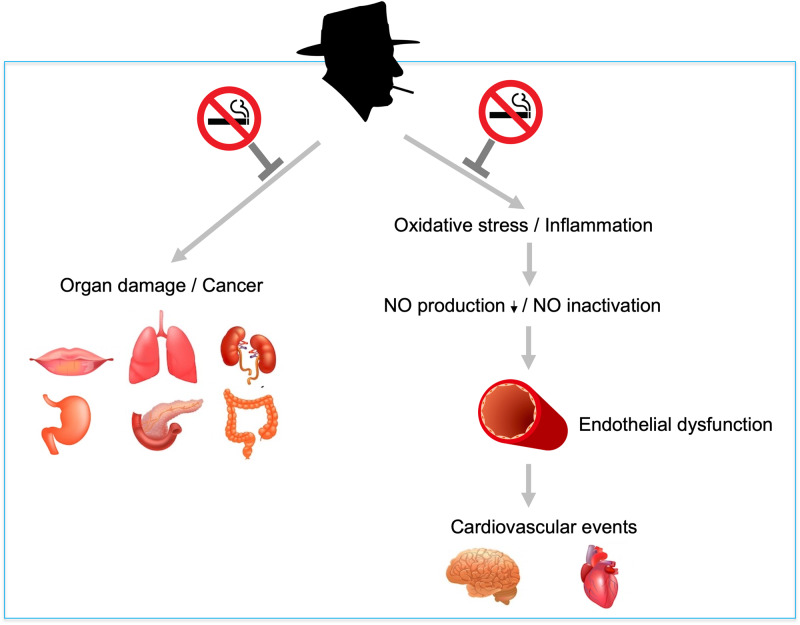
Smoking impairs vascular endothelial function and leads to atherosclerosis. Smoking cessation is expected to improve vascular endothelial function. Effects of smoking cessation on endothelial function are not always consistent. Further studies are needed to determine whether smoking cessation directly improves endothelial function. NO indicates nitric oxide.

Smoking impairs vascular endothelial function and leads to atherosclerosis. Smoking cessation is expected to improve vascular endothelial function. Effects of smoking cessation on endothelial function are not always consistent. Further studies are needed to determine whether smoking cessation directly improves endothelial function. NO indicates nitric oxide.

## Abstract (web summary)

Smoking impairs vascular endothelial function and leads to atherosclerosis. Smoking cessation is expected to improve vascular endothelial function. Effects of smoking cessation on endothelial function are not always consistent. Further studies are needed to determine whether smoking cessation directly improves endothelial function. NO indicates nitric oxide.

## Introduction

Smoking is deeply involved in the development, maintenance, and progression of atherosclerosis, and smoking itself is an independent risk factor for the development of cardiovascular events and death [1–4]. The clinical significance of vascular endothelial function has become clear in many aspects such as understanding the etiology, pathogenesis, involvement in the development of atherosclerosis, therapeutic targets, and prognostic factors. Vascular endothelial dysfunction is the first step in atherosclerosis and plays an important role in the development, maintenance, and progression of atherosclerosis, resulting in cardiovascular events [5, 6]. Smoking as well as hypertension, dyslipidemia, diabetes, obesity, aging, lack of exercise, excessive salt intake, and menopause are known to induce endothelial dysfunction [7]. Endothelial dysfunction can be improved by appropriate pharmacotherapy [8–11], replacement therapy [12, 13], and lifestyle modifications including aerobic exercise [14, 15], body weight reduction [16], decrease in salt intake [17] and smoking cessation [18–35]. The mechanisms by which smoking induces vascular endothelial dysfunction and the effects of smoking cessation on vascular endothelial function will be reviewed.

## Smoking and atherosclerosis

Epidemiological studies have shown a strong correlation between cardiovascular disease and the amount and duration of smoking [1]. Epidemiological studies and large clinical trials have indicated that smoking is a major risk factor for atherosclerosis [1–4]. Smoking is associated with elevated low-density lipoprotein cholesterol, decreased high-density lipoprotein cholesterol, increased blood catecholamine levels, increased fibrinogen levels, and impaired vascular endothelial function [36–48]. Although it is not clear whether smoking cessation can improve advanced atherosclerosis, it is expected to reverse the promotion of atherosclerosis caused by smoking. Indeed, among female smokers, two years of smoking cessation reduced cardiovascular mortality by 24% [49], and smoking cessation after myocardial infarction has been reported to reduce mortality by 20–50% compared with that in continuing smokers [50]. Regardless of smoking quantity, duration, or age at the start of smoking cessation, smoking cessation for about five years reduces the risk of developing cardiovascular events to that of never-smokers [2].

## Vascular endothelium structure and physiological function

The vascular endothelium is the innermost layer of the heart lumen, arteries, veins, and lymphatics and is composed of a single layer of vascular endothelial cells. Vascular endothelial cells produce and secrete many physiologically active substances including vasodilators, such as nitric oxide (NO), prostaglandin I_2_, C-type natriuretic peptide, and endothelium-derived vascular hyperpolarizing factor, and vasoconstrictors, such as endothelin, angiotensin II, prostaglandin H_2_ and thromboxane A_2_ [51, 52]. NO plays a very important role in atherosclerosis. The normal vascular endothelium regulates vasodilation and vasocontraction, proliferation and antiproliferation of vascular smooth muscle cells, coagulation and anticoagulation, inflammation and anti-inflammation, and oxidation and antioxidant effects, which work in balance to regulate and maintain vascular tone and structure [53]. Atherosclerosis develops as the first stage of vascular endothelial dysfunction. Further progression leads to cardiovascular events (e.g., angina pectoris, myocardial infarction, heart failure, stroke, and heart failure). If the entire body’s vascular endothelium could be collected, the total weight would be equivalent to that of the liver, and if it could be spread over an entire surface, the total area would be equivalent to six tennis courts, and it would be 100,000 km or two and a half times around the earth if it could be connected in a single row [6].

## Smoking and vascular endothelial function

Many studies have shown that the vascular endothelium-dependent relaxation response is impaired in smokers and in experimental animal models [42–48, 54, 55]. In our studies also, endothelial function was impaired in smokers compared to that in nonsmokers after forearm artery administration of acetylcholine, an agonist of NO production [46]. Pre-administration of the NO synthase (NOS) inhibitor N^G^-monomethyl-L-arginine acetate abolished the difference in endothelium-dependent relaxation induced by acetylcholine between smokers and nonsmokers [46]. In a study in which vasodilation induced by reactive hyperemia, flow-mediated vasodilation (FMD), in forearm arteries was investigated, it was shown that FMD was decreased in smokers and that FMD decreased with increase in the amount of smoking [20, 23–27, 31–33, 42, 48]. In coronary arteries, decreased reactivity to endogenous NO and decreased production of endogenous NO itself have also been noted in smokers [56]. It has been reported that vascular endothelial function is impaired in passive smokers as well as in smokers and that there is a correlation between passive smoking duration and endothelial dysfunction [57, 58]. In addition, smoking further exacerbates endothelial dysfunction in individuals with hypertension, diabetes, dyslipidemia, and other coronary risk factors [45]. In a multivariate analysis using FMD as the index, smoking was also confirmed to be an independent risk factor for vascular endothelial dysfunction [59].

Several studies have shown that endothelial function assessed by biomarkers for endothelial function including circulating levels of nitrate/nitrite, NO, von Willebrand factor (vWF), cellular adhesion molecules, vascular cell adhesion molecule-1 (VCAM-1) and intercellular adhesion molecule-1 (ICAM-1), endothelial progenitor cells (EPCs) and endothelial microparticles (EMPs), endothelin-1 and activity of endothelial NOS (eNOS) other than physiological assessment for endothelial function was impaired in smokers. These findings suggest that smoking is associated with endothelial dysfunction [17, 18, 21, 22, 29, 60–63].

## Mechanisms of smoking-induced vascular endothelial dysfunction

**Fig. 1:**
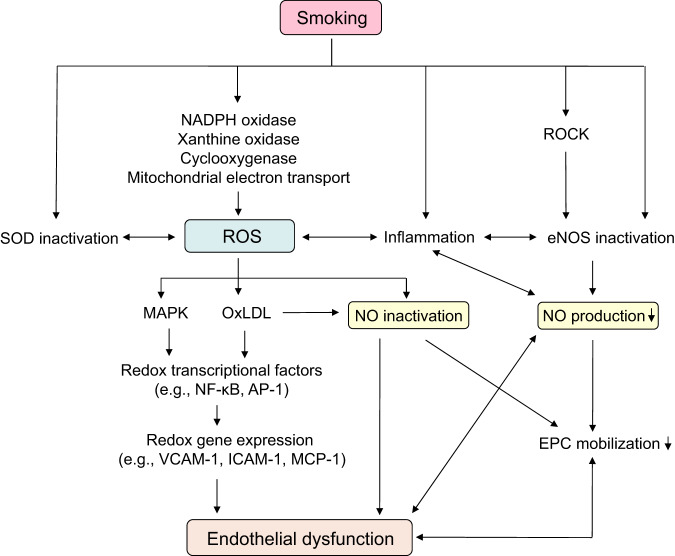
Putative mechanism of vascular endothelial dysfunction induced by smoking. NADPH indicates nicotinamide adenine dinucleotide phosphate, ROCK Rho-associated kinase, SOD superoxide dismutase, ROS reactive oxygen species, eNOS endothelial nitric oxide synthase, MAPK mitogen-activated protein kinase, oxLDL oxidative low-density lipoprotein, NO nitric oxide, NF-κB nuclear factor-kappa B, AP-1 activator protein-1, VCAM-1 vascular cell adhesion molecule-1, ICAM-1 intracellular adhesion molecule-1, MCP-1 macrophage chemotactic protein 1, EPC endothelial progenitor cell

Figure 1 shows the putative mechanisms of smoking-induced vascular endothelial dysfunction.

### Involvement of oxidative stress

The increase in reactive oxygen species (ROS) caused by smoking is thought to play a critical role in vascular endothelial dysfunction. Inactivation of NO due to the increased NO scavenging associated with increased ROS production has attracted attention as one of the mechanisms of vascular endothelial dysfunction [64–67]. The ROS produced are converted to peroxynitrite, which has a very strong cytotoxic effect when combined with NO, resulting in direct damage to vascular wall cells and a decrease in the biological activity of NO in vascular endothelial cells and vascular smooth muscle cells [68]. Oxidative stress conditions lead to vascular endothelial dysfunction, expression of redox-sensitive genes, induction of inflammation, and development of atherosclerosis [69]. This chain of processes leading to ROS overproduction and vascular endothelial damage exists not only in experimental animal models but also in humans [70]. Cigarette smoke contains a wide variety and large amount of ROS. In smokers, there is a decrease in blood ascorbic acid levels and an increase in various oxidative stress markers in blood or urine. Even single smoking decreases blood NO metabolites and antioxidants such as ascorbic acid, cysteine, methionine and uric acid [71]. In smokers, there appears to be a disruption of antioxidant mechanisms and an increase in ROS-producing systems [72]. Although there is no consensus regarding the increased activation of nicotinamide adenine dinucleotide phosphate oxidase by smoking, xanthine oxidase, an ROS-producing system, is activated by smoking. In smokers, a single dose of the xanthine oxidase inhibitor allopurinol restores the acetylcholine-induced endothelium-dependent relaxation response in forearm arteries to nonsmoker levels but does not affect the endothelium-dependent relaxation response in nonsmokers [73]. Rapid administration of the antioxidant vitamin C may improve endothelial function in smokers [74]. Deficiency of tetrahydrobiopterin, an essential cofactor for eNOS, has been shown to promote eNOS unpairing, which attenuates NO production and tends to enhance ROS production [75]. These basic clinical findings suggest that increased oxidative stress caused by smoking may contribute to vascular endothelial function. Increased oxidative stress due to smoking contributes to atherosclerosis by inducing cell proliferation, cell hypertrophy, and apoptosis, either directly through vascular endothelial damage or through activation of various intracellular signaling pathways. Furthermore, the onset of atherosclerosis feeds back to itself and aggravates the condition, making a vicious cycle that leads to the maintenance and progression of atherosclerosis.

### Involvement of Rho-associated kinase (ROCK)

The Rho family (Pho, Cdc42, Rac, and Rnd) belongs to the Ras superfamily of low molecular weight G proteins and is a major regulator of the actin cytoskeleton [76–80]. It is essential for vascular smooth muscle contraction, cell adhesion, cell death, cell division, and other processes of vascular formation. Furthermore, the RhoA/ROCK pathway plays an important role in the onset, maintenance, and progression of vascular injury through vasoconstriction and remodeling [81, 82]. It has been reported that plasminogen activator inhibitor-1 gene expression in endothelial cells and vascular remodeling are normalized by ROCK inhibitor treatment in atherosclerosis models [83, 84]. We confirmed that ROCK was activated in smokers and that ROCK activity was significantly correlated with FMD in smokers [85–87]. Although the detailed mechanism of smoking-induced activation of ROCK is not clear, smoking is thought to be directly involved in Rho activation. ROCK activation is known to be directly associated with decreased eNOS activity through reduced eNOSmRNA stability and inhibition of Akt phosphorylation [88]. Smoking-induced ROCK activation is associated with decreased eNOS activity and making a vicious cycle that may lead to further vascular endothelial dysfunction.

## Smoking cessation and vascular endothelial function

Unfortunately, there is little information on the effects of smoking cessation on endothelial function. Even taking into account publication bias, the number of published papers is very small. This may be due in part to the difficulty of smoking cessation. From 1998 to 2022, there were 23 studies in which the effects of smoking cessation on endothelial function in smokers were evaluated (Table 1) [18–35, 89–93]. Many studies including our studies have clearly shown that smoking is associated with endothelial dysfunction [42–48]. Therefore, it is strongly expected that smoking cessation will improve endothelial function. However, not all of the studies showed beneficial effects of smoking cessation on endothelial function. There were three studies using a prospective, double-blind, randomized, and placebo-control design [22, 89, 90]. Johnson et al. [22]. Showed that smoking cessation for one year improved FMD in the brachial artery in 1504 cigarette smokers. Mah et al. [89, 90]. Showed in two studies that smoking cessation alone did not improve FMD in healthy young cigarette smokers during smoking cessation for 7 days (*n* = 135) and for 24 h (*n* = 12), while smoking cessation with γ-tocopherol-rich supplementation improved FMD in both studies. Although 12 of 15 studies in which the effects of smoking cessation on vascular endothelial function was assessed by physiological methods including measurements of FMD and reactive hyperemia index (RHI) showed that smoking cessation improved FMD, RHI or vascular response to vasoactive agents [18, 21, 22, 25–29, 32–35], three of those 15 studies showed no effects of smoking cessation on FMD or RHI [89–91]. In eight studies in which the effects of smoking cessation on endothelial function were assessed by biomarkers[19, 20, 23, 24, 30, 31, 92, 93], five studies showed that smoking cessation increased or improved biomarkers for endothelial function including circulating levels of nitrate/nitrite, NO, vvWF, ICAM-1, EPCs and EMPs and activity of eNOS [19, 23, 24, 30, 31]. However, three of the eight studies showed no effects of smoking cessation on biomarkers for endothelial function [20, 92, 93]. In any case, there has been no report of smoking cessation worsening endothelial function.

**Table 1:** Effect of smoking cessation on endothelial function

Smoking cessation method	Publication year	Subjects (number)	Smoking cessation period	Endothelial function test	Results	Reference number
Nicotine nasal spray	1998	Cigarette smokers (*n* = 32)	7 days	NOx level	Did not alter circulating NOx levels.	Miller et al. [92]
Self-serve	1998	Heavy cigarette smokers (*n* = 7)	24 hours	BK-induced vasodilation	Improved BK-induced vasorelaxation in the dorsal hand vein.	Moreno et al. [18]
Nicotine replacement/self-serve	1999	Cigarette smokers with CV risks (*n* = 6)	12 weeks	FMD	Did not alter FMD in the brachial artery.	Jodoin et al. [91]
Nicotine replacement/self-serve	2004	Healthy cigarette smokers (*n* = 15)	4 weeks	EPC number	Increased the number of EPCs.	Kondo et al. [19]
Bupropion/nicotine replacement	2007	Cigarette smokers with CVD or CV risks (*n* = 41)	1 year	ICAM-1/vWF level	Decreased plasma ICAM-l levels but not vWF levels.	Halvorsen et al. [20]
Self-serve	2008	Cigarette smokers with recent MI (*n* = 35)	24 weeks	ACh-induced vasodilation	Improved ACh-induced vasorelaxation in the coronary artery.	Hosokawa et al. [21]
Nicotine replacement	2010	Cigarette smokers (*n* = 1504)	1 year	FMD	Improved FMD in the brachial artery.	Johnson et al. [22]
Nicotine replacement	2011	Cigarette smokers without CV risks (*n* = 122)	8 weeks	vWF level	Decreased circulating vWF levels.	Coponnetto et al. [23]
Nicotine replacement/self-serve	2011	Cigarette smokers (*n* = 144)	5 weeks	EPC number and function	Improved the function of EPCs but did not alter the number of EPCs.	Puls et al. [24]
Varenicline	2013	Cigarette smokers (*n* = 22)	12 weeks	FMD	Improved FMD in the brachial artery.	Umeda et al. [24]
Self-serve	2013	Cigarette smokers (*n* = 11)	8 weeks	FMD/RHI	Improved FMD in the brachial artery and RHI in the fingertip.	Sugiura et al. [25]
Placebo	2013	Healthy young cigarette smokers (*n* = 135)	7 days	FMD	Did not alter FMD in the brachial artery.	Mah et al. [89]
Varenicline	2014	Healthy cigarette smokers (*n* = 11)	12 weeks	FMD	Improved FMD in the brachial artery.	Kato et al. [27]
Nicotine replacement	2015	Healthy young cigarette smokers (*n* = 12)	24 hours	FMD	Did not alter FMD in the brachial artery.	Mah et al. [90]
Varenicline	2015	Cigarette smokers (*n* = 72)	20 weeks	FMD	Improved FMD in the brachial artery.	Kobayashi et al. [28]
Nicotine replacement	2016	Cigarette smokers (*n* = 34)	10±5 days	FMD	Improved FMD in the brachial artery.	Taylor et al. [29]
Varenicline	2016	Cigarette smokers with COPD (*n* = 18)	1 year	EMP level	Did not alter circulating EMPs levels.	Strulovici-Barel et al. [93]
Nicotine replacement	2017	Cigarette smokers with IDC (*n* = 153)	24 weeks	NO level/eNOS expression/eNOS activity	Increased NO level, eNOS expression and eNOS activity.	Wang et al. [30]
Varenicline/nicotine replacement	2017	Cigarette smokers (*n* = 188)	12 weeks	Endothelial glycocalyx	Restored endothelial glycocalyx.	Ikonomidis et al. [31]
Nicotine replacement	2019	Healthy cigarette smokers (*n* = 100)	12 weeks	RHI	Improved RHI in the fingertip.	Xue et al. [32]
Nicotine replacement/e-cigarette	2021	Cigarette smokers (*n* = 248)	3 days	FMD/ACh-induced CVC	Improved FMD in the brachial artery and ACh-induced CVC.	Klonizakis et al. [33]
Varenicline/nicotine replacement	2021	Cigarette smokers (*n* = 58)	20 weeks	FMD/RHI	Improved FMD in the brachial artery but did not alter RHI in the fingertip.	Fukumoto et al. [34]
Nicotine replacement/e-cigarette	2022	Cigarette smokers (*n* = 248)	12 weeks	FMD/ACh-induced CVC	Improved FMD in the brachial artery and ACh-induced CVC.	Klonizakis et al. [25]

*NOx* nitrate/nitrite, *BK* bradykinin, *FMD* flow-mediated vasodilation, *EPC* endothelial progenitor cell, *CV* cardiovascular, *CVD* cardiovascular disease, *ICAM-1* intercellular adhesion molecule-1, *vWF* von Willebrand factor, *MI* myocardial infraction, *Ach* acetylcholine, *EMP* endothelial microparticles, *IDC* ischemic dilated cardiomyopathy, *NO* nitric oxide, *eNOS* endothelial nitric oxide synthase, *RHI* reactive hyperemia index, *CVC* cutaneous vascular conductance

The reasons of the discrepant results of studies remain unclear. Depending on the degree of vascular endothelial dysfunction caused by smoking, smoking cessation is expected to improve vascular endothelial function as long as the impairment is not irreversible. Indeed, although many studies have clearly shown that several interventions including pharmacological therapy and lifestyle modifications improve endothelial function under the condition of relatively mild vascular endothelial dysfunction [8–35], we have shown that advanced endothelial dysfunction is not reversible even by appropriate pharmacological therapy [94]. Daily smoking amount, duration of smoking, duration of cessation, and background of individuals may play a critical role in the impact of smoking cessation on endothelial function. The method used for quitting smoking may also be important. The use of nicotine, bupropion, varenicline and e-cigarettes as an aid in smoking cessation may result in alteration of endothelial function. There were 15 studies on the use of nicotine replacement [19, 20, 22–24, 29–35, 90–92], and four of 14 studies showed that smoking cessation did not alter circulating NOx levels [92], improved the function of EPCs but not the number of EPCs [24], did not improve FMD [91], and improved FMD but not RHI [34]. In two of six studies on the use of varenicline, smoking cessation did not improve endothelial function [34, 93]. In only one study on the use of bupropion, smoking cessation decreased plasma ICAM-l levels but not vWF levels [20]. It has been shown that e-cigarettes per se has harmful effects on endothelial function [95, 96]. However, in both of the two studies on the use of e-cigarettes in combination with nicotine replacement, smoking cessation improved vascular function [33, 35]. In addition, effects of smoking cessation on endothelial function may differ by sex, age, and risk severity of the subject. Further studies are needed to confirm the effects of smoking cessation on endothelial function using an appropriate study design, several smoking cessation methods, short- to long-term follow-up periods, different grades of smoking status, and diverse subjects including subjects with advanced atherosclerosis and healthy subjects in large clinical trials.

## Conclusions

It is clear that smoking impairs vascular endothelial function and leads to atherosclerosis. Smoking cessation is expected to improve vascular endothelial function. However, the effects of smoking cessation on vascular endothelial function are not always consistent. In addition, the number of studies on the relationship between smoking cessation and vascular endothelial function is overwhelmingly small. Further studies are needed to elucidate the detailed mechanisms by which smoking induces endothelial dysfunction and to determine whether smoking cessation directly improves endothelial function.

### Supplementary information

Checklist
