# Supplementary material for: Smoking cessation and vascular endothelial function
Source: Hypertens Res. 2023 Oct 12;46(12):2670–8. doi: 10.1038/s41440-023-01455-z (PMC10695829; doi:10.1038/s41440-023-01455-z)
Supplement: Supplementary file 1 — Checklist [file 41440_2023_1455_MOESM1_ESM.docx]

**Hypertension Research: Checklist for Style**

To the Authors:

The following outline provides the order in which the necessary details for proper formatting in Hypertension Research. Please make sure that your manuscript follows all items before submission.

| **Title Page** | |
| --- | --- |
|  | Provide the information listed below:  - Title of the manuscript  (Editorial office recommends considering more attractive title that appeal your research findings.)  - Full names (First/last name and other initials) and academic affiliations of authors  - The corresponding author's name and e-mail address. |
| **Abstract** | |
|  | Abstract should be unstructured (without subheadings). |
|  | Supply 3 to 5 keywords below the Abstract. |
| **Text** | |
|  | Delete Revision history or Track Changes and change a text color to Black for not only the main document but also other file(s) such as Table or Figure if exist. |
| **Disclosure** | |
|  | Confirm your acknowledgements section is in the format below.   - Acknowledgements - Source of funding - Conflict of interest |
| **Reference** | |
|  | Make sure that the reference style follows the Guide for Authors (if there are more than six authors, list the first six authors followed by et al.), and confirm the references to the literature should be numbered consecutively and given in brackets like [n].  **Example of references** (Journal article)  Glodny B, Pauli G. Medullopressin: a new pressor activity from the renal medulla. Hypertens Res. 2005; 28: 827–36. |

| **Tables and Figures** | |
| --- | --- |
|  | Figures or Tables should not be inserted in the main text and supplied as separate files each. |
|  | Tables should be supplied as editable Word or Excel file formats. |
| **Figures** | |
|  | Make sure that Figures are numbered on image and labeled sequentially as Figure 1, Figure 2, etc. |
|  | Supply the Figure legend separately from the figures, and include it in the manuscript text (after the reference list). |
|  | ALL figures should be of a high enough quality to be assessed in the peer review process. |
| **Supplementary Information** | |
|  | Supplemental data must use the format “Supplementary Figure 1”, “Supplementary Table 1” etc. |
| **English proofreading** | |
|  | Confirm that your manuscript is proofread by a professional English-native or English editing service. |

We appreciate your understanding and kind cooperation!
